# *tert*-Butyl 2-(4-chloro­benzo­yl)-2-methyl­propanoate

**DOI:** 10.1107/S1600536810003156

**Published:** 2010-01-30

**Authors:** Chelsey M. Crosse, Emily C. Kelly, Marshall W. Logue, Rudy L. Luck, John S. Maass, Katlyn C. Mehne, Louis R. Pignotti

**Affiliations:** a Department of Chemistry, 1400 Townsend Drive, Michigan Technological University, Houghton, MI 49931, USA

## Abstract

The title compound, C_15_H_19_ClO_3_, is bent with a dihedral angle of 72.02 (9)° between the mean planes of the benzene ring and a group encompassing the ester functionality (O=C—O—C). In the crystal, mol­ecules related by inversion symmetry are connected by weak C—H⋯O inter­actions into infinite chains. These inter­actions involve H atoms from a methyl group of the dimethyl residue and the O atoms of the ketone on one side of a mol­ecule; on the other side there are inter­actions between H atoms of the benzene ring and the carbonyl O atoms of the ester functionality. There are no directional inter­actions between the chains.

## Related literature

For the synthesis, spectroscopic characterization and reactivity of the title compound, see: Logue (1974 ▶); Logue *et al.* (1975 ▶). For related structures, see: Crosse *et al.* (2010 ▶); Gould *et al.* (2010 ▶); Logue *et al.* (2010 ▶). For the syntheses and characterization of structurally similar indanone-derived β-keto ester derivatives, see: Mouri *et al.* (2009 ▶); Noritake *et al.* (2008 ▶); Rigby & Dixon (2008 ▶). For weak hydrogen-bonded inter­actions, see: Karle *et al.* (2009 ▶). 
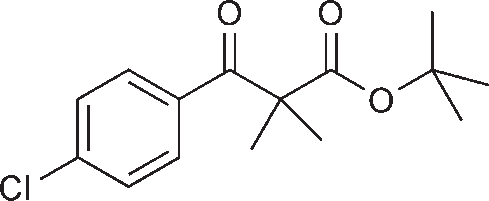


## Experimental

### 

#### Crystal data

C_15_H_19_ClO_3_
*M* *_r_* = 282.75
Triclinic, 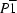

*a* = 8.601 (3) Å
*b* = 9.214 (2) Å
*c* = 11.033 (2) Å
α = 72.67 (2)°
β = 74.62 (2)°
γ = 74.02 (3)°
*V* = 786.3 (4) Å^3^
*Z* = 2
Mo *K*α radiation
μ = 0.24 mm^−1^
*T* = 291 K
0.30 × 0.30 × 0.30 mm

#### Data collection

Enraf–Nonius TurboCAD-4 diffractometer
Absorption correction: ψ scan (North *et al.*, 1968 ▶) *T*_min_ = 0.905, *T*_max_ = 0.929
2961 measured reflections
2759 independent reflections
1589 reflections with *I* > 2σ(*I*)
*R*_int_ = 0.020
3 standard reflections every 166 min  intensity decay: 9%

#### Refinement

*R*[*F* ^2^ > 2σ(*F* ^2^)] = 0.047
*wR*(*F* ^2^) = 0.125
*S* = 1.01
2759 reflections
177 parameters
H-atom parameters constrained
Δρ_max_ = 0.16 e Å^−3^
Δρ_min_ = −0.17 e Å^−3^

### 

Data collection: *CAD-4 EXPRESS* (Enraf–Nonius, 1994 ▶); cell refinement: *CAD-4 EXPRESS*; data reduction: *XCAD4* (Harms & Wocadlo, 1995 ▶); program(s) used to solve structure: *SIR2004* (Burla *et al.*, 2005 ▶); program(s) used to refine structure: *SHELXL97* (Sheldrick, 2008 ▶); molecular graphics: *ORTEP-3 for Windows* (Farrugia, 1997 ▶) and *Mercury* (Macrae *et al.*, 2008 ▶); software used to prepare material for publication: *WinGX* (Farrugia, 1999 ▶) and *publCIF* (Westrip, 2010 ▶).

## Supplementary Material

Crystal structure: contains datablocks global, I. DOI: 10.1107/S1600536810003156/zl2267sup1.cif

Structure factors: contains datablocks I. DOI: 10.1107/S1600536810003156/zl2267Isup2.hkl

Additional supplementary materials:  crystallographic information; 3D view; checkCIF report

## Figures and Tables

**Table 1:** Hydrogen-bond geometry (Å, °)

*D*—H⋯*A*	*D*—H	H⋯*A*	*D*⋯*A*	*D*—H⋯*A*
C10—H10*A*⋯O1^i^	0.96	2.58	3.476 (4)	155
C5—H5⋯O2^ii^	0.93	2.7	3.316 (3)	125

Symmetry codes: (i) 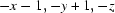; (ii) 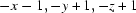.

**Fig. 1.**
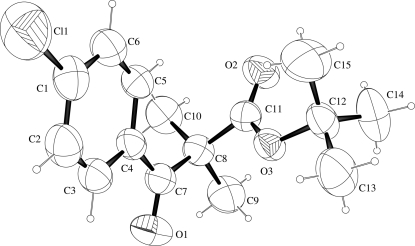
ORTEP-3 (Farrugia, 1997) drawing of the title compound with displacement ellipsoids drawn at the 50% probability level.

**Fig. 2.**
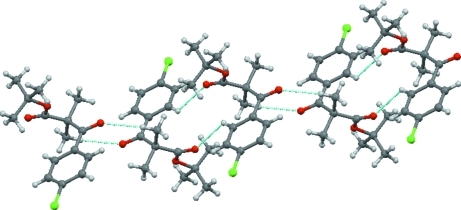
A Mercury (Macrae et al., 2008) illustration of the packing in the title compound depicting the H-bonded linkages using dashed lines which result in infinite chains.

## supplementary crystallographic information

### Comment

Treatment of 2,2-disubstituted *t*-butyl β-keto esters with trifluoroacetic acid at room temperature quantitatively generates the corresponding 2,2-disubstituted β-keto acids, which were used to probe the nature of the transition state for the thermal decarboxylation of β-keto acids (Logue *et al.*, 1975).  Structurally similar indanone-derived β-keto ester derivatives have been prepared recently (Mouri *et al.*, 2009; Noritake *et al.*, 2008; Rigby & Dixon, 2008). The directing nature of weak C—H···O H-bonds has been noted to be of importance to afford the three dimensional structure observed in these kinds of molecules (Karle *et al.*, 2009).

In this contribution we present the solid state structure of one such 2,2-disubstituted β-keto acid, i.e. the title compound being the *para*-chlorobenzene derivative. This is the third paper in a series of four dealing with substituted derivatives (H–, CH_3_–, Cl- (this paper) and NO_2_– on the *para*-position of the phenyl ring) of the title compound. A more detailed comparison of all four substitution compounds will be given in the fourth paper of this series (Crosse *et al.*, 2010).

The molecule, Fig. 1, displays a bent geometry with a dihedral angle between the mean planes of the phenyl ring and a plane composed of the ester functionality of 72.02 (9)°. Molecules are linked by C—H···O weak hydrogen bonds generating infinite chains as shown in Fig. 2. The phenyl rings are not involved in intercalation or stacking interactions either within or between the chains. Instead, neighbouring *t*-butyl groups on adjacent chains exhibit hydrophobic stacking.

### Experimental

Crystals of the material were synthesized as reported earlier and were grown by evaporation of a solution in hexane (Logue, 1974). *M*.p. 600–606 K. IR (neat, cm^-1^) 2982 (m, ring), 1726 (s, C=O), 1682 (s, O—C=O), 1588 (*s*), 1488 (*m*), 1388 (*m*), 1368 (w), 1273 (*m*), 1133 (*m*), 1091.6 (*m*), 987 (w), 920 (w), 842 (*m*), 739 (*m*). ^1^H NMR (CDCl_3_) δ: 1.27 (s, 9*H*), 1.47 (s, 6*H*), 7.36 (d, 2*H*, J=8.8 Hz), 7.80 (d, 2*H*, J=9.2 Hz). ^13^C NMR (CDCl_3_) δ: 23.8, 27.6, 54.0, 81.9, 130.2, 133.6, 139.0, 173.8, 196.8.

### Refinement

All H atoms were placed at calculated positions, with C—H = 0.93 Å (aromatic) or 0.96 Å (methyl) and refined using a riding model with *U*_iso_(H) constrained to be 1.5 *U*_eq_(C) for methyl groups and 1.2 *U*_eq_(C) for all other C atoms. The quality of the data as reflected by only 58% of the reflections observed, large ADP's and inaccurate C—C bond lengths is low. The data had been collected on a 30 year old single point detector instrument not equipped with a low temperature device as part of a class project with undergraduate students. Due to the time constraints imposed by the class schedule a maximum exposure time of 60 s had to be alloted for measuring each reflection.

### Crystal data

**Table:**

C_15_H_19_ClO_3_	*Z* = 2
*M**_r_* = 282.75	*F*(000) = 300
Triclinic, *P*1	*D*_x_ = 1.194 Mg m^−^^3^
Hall symbol: -P 1	Mo *K*α radiation, λ = 0.71073 Å
*a* = 8.601 (3) Å	Cell parameters from 25 reflections
*b* = 9.214 (2) Å	θ = 10–15°
*c* = 11.033 (2) Å	µ = 0.24 mm^−^^1^
α = 72.67 (2)°	*T* = 291 K
β = 74.62 (2)°	Prism, colourless
γ = 74.02 (3)°	0.30 × 0.30 × 0.30 mm
*V* = 786.3 (4) Å^3^	

### Data collection

**Table:**

Enraf–Nonius TurboCAD-4 diffractometer	1589 reflections with *I* > 2σ(*I*)
Radiation source: Enraf Nonius FR590	*R*_int_ = 0.020
graphite	θ_max_ = 25.0°, θ_min_ = 2.0°
non–profiled ω/2τ scans	*h* = 0→10
Absorption correction: ψ scan (North *et al.*, 1968)	*k* = −10→10
*T*_min_ = 0.905, *T*_max_ = 0.929	*l* = −12→13
2961 measured reflections	3 standard reflections every 166 min
2759 independent reflections	intensity decay: 9%

### Refinement

**Table:**

Refinement on *F*^2^	Primary atom site location: structure-invariant direct methods
Least-squares matrix: full	Secondary atom site location: difference Fourier map
*R*[*F*^2^ > 2σ(*F*^2^)] = 0.047	Hydrogen site location: inferred from neighbouring sites
*wR*(*F*^2^) = 0.125	H-atom parameters constrained
*S* = 1.00	*w* = 1/[σ^2^(*F*_o_^2^) + (0.0499*P*)^2^ + 0.1997*P*] where *P* = (*F*_o_^2^ + 2*F*_c_^2^)/3
2759 reflections	(Δ/σ)_max_ < 0.001
177 parameters	Δρ_max_ = 0.16 e Å^−^^3^
0 restraints	Δρ_min_ = −0.17 e Å^−^^3^

### Special details

**Table:**

Experimental. Number of psi-scan sets used was 3. Theta correction was applied. Averaged transmission function was used. No Fourier smoothing was applied.
Geometry. All s.u.'s (except the s.u. in the dihedral angle between two l.s. planes) are estimated using the full covariance matrix. The cell s.u.'s are taken into account individually in the estimation of s.u.'s in distances, angles and torsion angles; correlations between s.u.'s in cell parameters are only used when they are defined by crystal symmetry. An approximate (isotropic) treatment of cell s.u.'s is used for estimating s.u.'s involving l.s. planes.
Refinement. Refinement of *F*^2^ against ALL reflections. The weighted *R*-factor *wR* and goodness of fit *S* are based on *F*^2^, conventional *R*-factors *R* are based on *F*, with *F* set to zero for negative *F*^2^. The threshold expression of *F*^2^ > 2σ(*F*^2^) is used only for calculating *R*-factors(gt) *etc*. and is not relevant to the choice of reflections for refinement. *R*-factors based on *F*^2^ are statistically about twice as large as those based on *F*, and *R*- factors based on ALL data will be even larger.

### Fractional atomic coordinates and isotropic or equivalent isotropic displacement parameters (Å^2^)

**Table:**

	*x*	*y*	*z*	*U*_iso_*/*U*_eq_	
Cl1	0.16763 (12)	0.07087 (11)	0.34258 (10)	0.1148 (4)	
C1	0.0088 (3)	0.2115 (3)	0.2795 (3)	0.0715 (8)	
C2	0.0200 (4)	0.2567 (3)	0.1470 (3)	0.0732 (8)	
H2	0.112	0.2139	0.0922	0.088*	
C3	−0.1066 (3)	0.3656 (3)	0.0975 (3)	0.0652 (7)	
H3	−0.0996	0.3952	0.0083	0.078*	
C4	−0.2453 (3)	0.4331 (3)	0.1768 (2)	0.0539 (6)	
C5	−0.2524 (3)	0.3851 (3)	0.3101 (2)	0.0616 (7)	
H5	−0.3433	0.4288	0.3654	0.074*	
C6	−0.1270 (4)	0.2740 (3)	0.3615 (3)	0.0733 (8)	
H6	−0.1341	0.2417	0.4507	0.088*	
C7	−0.3731 (3)	0.5568 (3)	0.1130 (2)	0.0567 (6)	
O1	−0.3574 (2)	0.5870 (2)	−0.00407 (18)	0.0818 (6)	
C8	−0.5234 (3)	0.6450 (3)	0.1932 (2)	0.0580 (7)	
C9	−0.6140 (4)	0.7842 (4)	0.1003 (3)	0.0858 (9)	
H9A	−0.6522	0.7464	0.0436	0.129*	
H9B	−0.5396	0.8509	0.0499	0.129*	
H9C	−0.7065	0.8416	0.1499	0.129*	
C10	−0.6413 (3)	0.5359 (4)	0.2725 (3)	0.0815 (9)	
H10A	−0.6754	0.4959	0.215	0.122*	
H10B	−0.7364	0.5926	0.3205	0.122*	
H10C	−0.5855	0.451	0.3316	0.122*	
C11	−0.4693 (3)	0.7139 (3)	0.2811 (3)	0.0557 (6)	
O2	−0.5376 (2)	0.7174 (2)	0.39021 (18)	0.0768 (6)	
O3	−0.3370 (2)	0.77357 (18)	0.21611 (15)	0.0573 (5)	
C12	−0.2559 (3)	0.8503 (3)	0.2752 (3)	0.0652 (7)	
C13	−0.1158 (4)	0.8952 (4)	0.1653 (3)	0.0982 (11)	
H13A	−0.1601	0.9655	0.0928	0.147*	
H13B	−0.0465	0.8035	0.14	0.147*	
H13C	−0.052	0.945	0.1934	0.147*	
C14	−0.3741 (4)	0.9938 (4)	0.3095 (4)	0.1056 (12)	
H14A	−0.4215	1.0563	0.2361	0.158*	
H14B	−0.3158	1.053	0.3331	0.158*	
H14C	−0.4602	0.9636	0.381	0.158*	
C15	−0.1904 (5)	0.7350 (4)	0.3885 (3)	0.1139 (13)	
H15A	−0.2809	0.7124	0.4587	0.171*	
H15B	−0.1196	0.7781	0.4159	0.171*	
H15C	−0.129	0.6409	0.3633	0.171*	

### Atomic displacement parameters (Å^2^)

**Table:**

	*U*^11^	*U*^22^	*U*^33^	*U*^12^	*U*^13^	*U*^23^
Cl1	0.0975 (7)	0.1088 (7)	0.1345 (8)	0.0243 (5)	−0.0444 (6)	−0.0478 (6)
C1	0.0677 (18)	0.0628 (18)	0.088 (2)	−0.0045 (14)	−0.0202 (16)	−0.0288 (16)
C2	0.0654 (18)	0.0720 (19)	0.080 (2)	−0.0162 (16)	0.0064 (16)	−0.0313 (16)
C3	0.0745 (19)	0.0649 (17)	0.0575 (16)	−0.0239 (15)	0.0015 (15)	−0.0214 (14)
C4	0.0614 (15)	0.0556 (15)	0.0498 (14)	−0.0241 (13)	−0.0005 (12)	−0.0191 (12)
C5	0.0671 (17)	0.0592 (16)	0.0559 (16)	−0.0098 (14)	−0.0034 (13)	−0.0213 (13)
C6	0.084 (2)	0.0683 (18)	0.0657 (17)	−0.0017 (16)	−0.0181 (16)	−0.0243 (15)
C7	0.0618 (17)	0.0631 (16)	0.0541 (16)	−0.0303 (14)	−0.0073 (13)	−0.0156 (13)
O1	0.0877 (14)	0.1077 (16)	0.0519 (12)	−0.0260 (12)	−0.0120 (10)	−0.0194 (11)
C8	0.0535 (15)	0.0655 (16)	0.0594 (15)	−0.0163 (13)	−0.0123 (12)	−0.0171 (13)
C9	0.081 (2)	0.093 (2)	0.090 (2)	−0.0075 (17)	−0.0387 (18)	−0.0230 (18)
C10	0.0607 (18)	0.104 (2)	0.090 (2)	−0.0384 (17)	0.0024 (15)	−0.0343 (18)
C11	0.0538 (15)	0.0572 (16)	0.0549 (16)	−0.0106 (13)	−0.0100 (13)	−0.0139 (12)
O2	0.0796 (13)	0.0971 (15)	0.0575 (12)	−0.0311 (11)	0.0045 (10)	−0.0289 (10)
O3	0.0587 (10)	0.0672 (11)	0.0543 (10)	−0.0232 (9)	−0.0074 (8)	−0.0217 (8)
C12	0.0684 (17)	0.0764 (18)	0.0660 (17)	−0.0271 (15)	−0.0107 (14)	−0.0314 (15)
C13	0.090 (2)	0.122 (3)	0.105 (3)	−0.058 (2)	0.0055 (19)	−0.049 (2)
C14	0.096 (3)	0.102 (3)	0.145 (3)	−0.026 (2)	−0.008 (2)	−0.077 (2)
C15	0.131 (3)	0.137 (3)	0.100 (3)	−0.054 (3)	−0.063 (2)	−0.009 (2)

### Geometric parameters (Å, °)

**Table:**

Cl1—C1	1.739 (3)	C9—H9C	0.96
C1—C6	1.376 (4)	C10—H10A	0.96
C1—C2	1.380 (4)	C10—H10B	0.96
C2—C3	1.370 (4)	C10—H10C	0.96
C2—H2	0.93	C11—O2	1.197 (3)
C3—C4	1.392 (3)	C11—O3	1.337 (3)
C3—H3	0.93	O3—C12	1.483 (3)
C4—C5	1.394 (3)	C12—C15	1.504 (4)
C4—C7	1.498 (4)	C12—C14	1.509 (4)
C5—C6	1.380 (4)	C12—C13	1.515 (4)
C5—H5	0.93	C13—H13A	0.96
C6—H6	0.93	C13—H13B	0.96
C7—O1	1.216 (3)	C13—H13C	0.96
C7—C8	1.536 (3)	C14—H14A	0.96
C8—C11	1.526 (3)	C14—H14B	0.96
C8—C10	1.540 (4)	C14—H14C	0.96
C8—C9	1.546 (4)	C15—H15A	0.96
C9—H9A	0.96	C15—H15B	0.96
C9—H9B	0.96	C15—H15C	0.96
			
C6—C1—C2	120.9 (3)	C8—C10—H10B	109.5
C6—C1—Cl1	120.0 (2)	H10A—C10—H10B	109.5
C2—C1—Cl1	119.0 (2)	C8—C10—H10C	109.5
C3—C2—C1	119.0 (3)	H10A—C10—H10C	109.5
C3—C2—H2	120.5	H10B—C10—H10C	109.5
C1—C2—H2	120.5	O2—C11—O3	125.4 (2)
C2—C3—C4	122.0 (3)	O2—C11—C8	125.1 (2)
C2—C3—H3	119	O3—C11—C8	109.5 (2)
C4—C3—H3	119	C11—O3—C12	122.39 (19)
C3—C4—C5	117.5 (3)	O3—C12—C15	109.6 (2)
C3—C4—C7	117.9 (2)	O3—C12—C14	109.8 (2)
C5—C4—C7	124.5 (2)	C15—C12—C14	113.7 (3)
C6—C5—C4	121.1 (2)	O3—C12—C13	101.9 (2)
C6—C5—H5	119.4	C15—C12—C13	110.8 (3)
C4—C5—H5	119.4	C14—C12—C13	110.4 (3)
C1—C6—C5	119.4 (3)	C12—C13—H13A	109.5
C1—C6—H6	120.3	C12—C13—H13B	109.5
C5—C6—H6	120.3	H13A—C13—H13B	109.5
O1—C7—C4	119.2 (2)	C12—C13—H13C	109.5
O1—C7—C8	119.7 (2)	H13A—C13—H13C	109.5
C4—C7—C8	121.1 (2)	H13B—C13—H13C	109.5
C11—C8—C7	110.7 (2)	C12—C14—H14A	109.5
C11—C8—C10	111.1 (2)	C12—C14—H14B	109.5
C7—C8—C10	110.0 (2)	H14A—C14—H14B	109.5
C11—C8—C9	106.4 (2)	C12—C14—H14C	109.5
C7—C8—C9	109.0 (2)	H14A—C14—H14C	109.5
C10—C8—C9	109.7 (2)	H14B—C14—H14C	109.5
C8—C9—H9A	109.5	C12—C15—H15A	109.5
C8—C9—H9B	109.5	C12—C15—H15B	109.5
H9A—C9—H9B	109.5	H15A—C15—H15B	109.5
C8—C9—H9C	109.5	C12—C15—H15C	109.5
H9A—C9—H9C	109.5	H15A—C15—H15C	109.5
H9B—C9—H9C	109.5	H15B—C15—H15C	109.5
C8—C10—H10A	109.5		

### Hydrogen-bond geometry (Å, °)

**Table:**

*D*—H···*A*	*D*—H	H···*A*	*D*···*A*	*D*—H···*A*
C10—H10A···O1^i^	0.96	2.58	3.476 (4)	155
C5—H5···O2^ii^	0.93	2.7	3.316 (3)	125

Symmetry codes: (i) −*x*−1, −*y*+1, −*z*; (ii) −*x*−1, −*y*+1, −*z*+1.
